# Polyamines promote xenobiotic nucleic acid synthesis by modified thermophilic polymerase mutants†

**DOI:** 10.1039/d4cb00017j

**Published:** 2024-04-04

**Authors:** Hidekazu Hoshino, Yuuya Kasahara, Satoshi Obika

**Affiliations:** a National Institutes of Biomedical Innovation, Health and Nutrition (NIBIOHN) 7-6-8 Saito-Asagi Ibaraki 567-0085 Osaka Japan h-hoshino@nibiohn.go.jp; b Graduate School of Pharmaceutical Sciences, Osaka University 1-6 Yamadaoka Suita 565-0871 Osaka Japan obika@phs.osaka-u.ac.jp

## Abstract

The enzymatic synthesis of xenobiotic nucleic acids (XNA), which are artificially sugar-modified nucleic acids, is essential for the preparation of XNA libraries. XNA libraries are used in the *in vitro* selection of XNA aptamers and enzymes (XNAzymes). Efficient enzymatic synthesis of various XNAs can enable the screening of high-quality XNA aptamers and XNAzymes by expanding the diversity of XNA libraries and adding a variety of properties to XNA aptamers and XNAzymes. However, XNAs that form unstable duplexes with DNA, such as arabino nucleic acid (ANA), may dissociate during enzyme synthesis at temperatures suitable for thermophilic polymerases. Thus, such XNAs are not efficiently synthesised by the thermophilic polymerase mutants at the end of the sequence. This undesirable bias reduces the possibility of generating high-quality XNA aptamers and XNAzymes. Here, we demonstrate that polyamine-induced DNA/ANA duplex stabilisation promotes ANA synthesis that is catalysed by thermophilic polymerase mutants. Several polyamines, including spermine, spermidine, cadaverine, and putrescine promote ANA synthesis. The negative effect of polyamines on the fidelity of ANA synthesis was negligible. We also showed that polyamines promote the synthesis of other XNAs, including 2′-amino-RNA/2′-fluoro-RNA mixture and 2′-*O*-methyl-RNA. In addition, we found that polyamine promotes DNA synthesis from the 2′-*O*-methyl-RNA template. Polyamines, with the use of thermophilic polymerase mutants, may allow further development of XNA aptamers and XNAzymes by promoting the transcription and reverse transcription of XNAs.

## Abstract 2

Polyamines stimulate the synthesis of various xenobiotic nucleic acids (XNA) by modified thermophilic DNA polymerase mutants.

## Graphical abstract

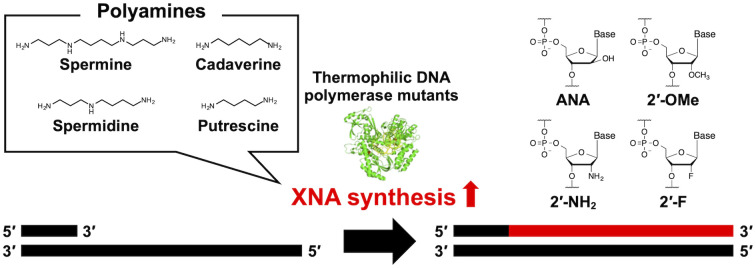

## Introduction

In the last few decades, many nucleic acid analogues, with different sugar backbones, have been synthesised. The nucleic acid analogues are referred to as xenobiotic nucleic acids (XNAs).^1,2^ Modification of the sugar moieties affects the nucleic acid properties such as nuclease resistance and duplex stability. Modification of the XNA properties allows for its use in therapeutics, such as in the development of antisense oligonucleotides (ASO), splice-switching oligonucleotides (SSO), small interfering RNA (siRNA), aptamers, nucleic acid enzymes, and CRISPR-RNAs (crRNAs) drugs.^3^

In the last decade, *in vitro* selection experiments have resulted in the development of XNA aptamers^4–12^ and XNAzymes.^13–15^ One of the significant advantages of XNA aptamers and XNAzymes is that they are nuclease resistant without post-modification. The DNA or RNA in screened aptamers need to be replaced with XNA for use in drugs.^9,16–18^ In contrast, XNA aptamers and XNAzymes screened from the XNA library are inherently nuclease resistant.^7,8^ Therefore, there is no need to risk reducing the activity of aptamers or nucleic acid enzymes by replacing DNA or RNA with XNA. Another significant advantage of XNA aptamers and XNAzymes is that the XNA libraries are highly diverse, increasing the likelihood of obtaining highly active XNA aptamers and XNAzymes. For example, introduction of new functional groups can create new interactions with target molecules.^19–21^

Polymerase mutants are required for the development of XNA aptamers and XNAzymes and are applied during transcription (DNA → XNA) and reverse transcription (XNA → DNA). XNA libraries can be enzymatically synthesised by polymerase mutants using DNA templates. Polymerase mutants, derived from thermophilic DNA polymerases such as Taq, 9°N, Tgo, and KOD DNA polymerase have been developed for XNA synthesis.^4,5,22–26^ Based on KOD DNA polymerase, we previously developed the polymerase mutant, KOD DGLNK (KOD: N210D/Y409G/A485L/D614N/E664K), for the synthesis of locked nucleic acid (LNA), also called 2′,4′-bridged nucleic acid (2′,4′-BNA).^5^

**Fig. 1:**
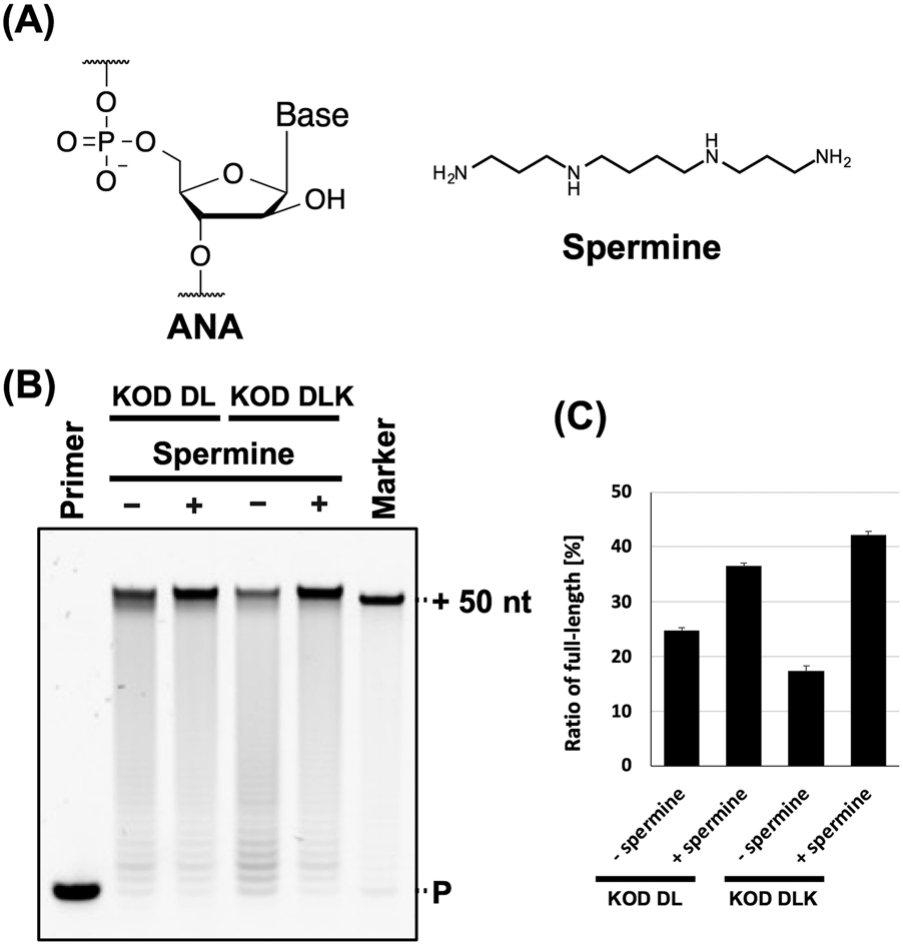
Promotion of ANA synthesis by spermine. (A) Chemical structure of ANA and spermine. (B) Denaturing PAGE analysis of ANA synthesis by KOD DL or KOD DLK with and without spermine. Reaction solution contains 1 × KOD Dash buffer, 1 mM MnSO_4_, 0.4 μM FAM-labeled DNA primer (25-mer, Primer#1), 0.6 μM DNA template (75-mer, Template#1_N50), 0.1 mM Ara-NTP, spermine (0 or 2 mM for KOD DL; and 0 or 5 mM for KOD DLK), and 20 ng μL^−1^ KOD DL or KOD DLK. ANA was synthesized at 60 °C for 5 min. P: primer. (C) Bar plot of the ratio of full-length product from the reaction shown in Fig. 1B. The ratio of full-length product was calculated from the fluorescence intensity. Data represent the mean and standard error of three independent experiments.

Some XNAs, such as arabino nucleic acid (ANA)^27^ (Fig. 1A), are not suitable for high-temperature reactions because of the instability of the DNA/XNA duplex. Lowering the reaction temperature to stabilise the DNA/XNA duplex reduces the thermostable polymerase activity. Therefore, the DNA/XNA duplex stability is an important factor in XNA synthesis. Here, we focused on polyamines. Polyamines are organic compounds with more than two amino groups. Cationic polyamines bind to DNA and RNA *via* electrostatic interactions and increase the nucleic acids duplex stability of both DNA/DNA and DNA/RNA duplexes,^28–30^ thereby promoting DNA and RNA synthesis.^31–33^ Therefore, in this study, we aimed to determine whether polyamines promote ANA synthesis. In addition, we aimed to evaluate the effects of polyamines on other XNAs (2′-amino-RNA (2′-NH_2_-RNA), 2′-fluoro-RNA (2′-F-RNA), and 2′-*O*-methyl-RNA (2′-OMe-RNA)) synthesis and reverse transcription, DNA synthesis from 2′-OMe-RNA template, by thermophilic polymerase mutants. Furthermore, we aimed to determine the effect of polyamines on the fidelity of ANA synthesis.

## Results and discussion

The stability of DNA/ANA duplexes is much lower than that of DNA/DNA duplexes.^27^ In addition, polyamines increase nucleic acid duplex stability. Therefore, we aimed to evaluate the effects of polyamines on ANA synthesis. First, we screened for polymerase mutants with ANA synthesis activity from the polymerase mutants we generated previously.^5^ The polymerase mutants KOD DL (KOD: N210D/A485L) and KOD DLK (KOD: N210D/A485L/E664K) were selected. N210D is an exonuclease-deficient mutation^34^ and A485L is an allosteric mutation that promotes the XNA synthesis.^35^ E664K can improve the binding affinity of the polymerase to the DNA/XNA duplex by substituting an anionic residue with a cationic residue.^4,36^ We evaluated the effects of the common polyamines spermine (Fig. 1A).^31,32^

ANA synthesis was evaluated by primer extension. Reaction solutions containing ANA triphosphates (Ara-ATP, Ara-GTP, Ara-CTP, and Ara-UTP), FAM-labelled DNA primer, and DNA templates containing 50-mer random sequences were prepared. Spermine and polymerase mutants were sequentially added to the solutions after annealing. Following ANA synthesis, synthesis efficiency was evaluated by performing denaturing polyacrylamide gel electrophoresis (PAGE). We found that spermine promoted ANA synthesis *via* KOD DL and KOD DLK (Fig. 1B). The optimal concentration of spermine for each polymerase was determined (Fig. S1A and B, ESI†). The reaction conditions and optimal polyamine concentrations are listed in Table 1. The effect of polyamines differed depending on the polymerase mutant used. ANA synthesis by KOD DLK was more strongly promoted by spermine than that by KOD DL (ANA synthesis efficiency: KOD DLK (+polyamine) > KOD DL (+polyamine) > KOD DL (−polyamine) > KOD DLK (−polyamine)) (Fig. 1C). This difference may be due to differences in optimal concentrations. The optimal concentrations of spermine for KOD DL and KOD DLK were 2 mM and 5 mM, respectively. KOD DLK was suitable for use with higher concentrations of spermine and may have benefited more from the polyamine effect and duplex stabilisation. The difference between KOD DL and KOD DLK was the mutation E664K, which promotes the binding affinity to the DNA/XNA duplex. Because both spermine and polymerase mutants interact with DNA/ANA duplexes, spermine and polymerase mutants compete for DNA/ANA duplexes. Therefore, we believe that KOD DLK, which has a stronger DNA/ANA duplex binding affinity, could tolerate higher concentrations of spermine, thereby synthesising ANA more efficiently.

**Table 1:** Reaction conditions and optimized concentration of polyamines for XNAs synthesis

Triphosphate	Polymerase	Reaction conditions	Optimized polyamine
ANA	20 ng μL^−1^ KOD DL	60 °C, 5 min	2 mM spermine
ANA	20 ng μL^−1^ KOD DLK	60 °C, 5 min	5 mM spermine
ANA	20 ng μL^−1^ KOD DLK	60 °C, 5 min	9 mM spermidine
ANA	20 ng μL^−1^ KOD DLK	60 °C, 5 min	13 mM cadaverine
ANA	20 ng μL^−1^ KOD DLK	60 °C, 5 min	14 mM putrescine
2′-NH_2_-RNA and 2′-F-RNA	300 ng μL^−1^ KOD DSLNK	55 °C, 90 min	2 mM spermine
2′-NH_2_-RNA and 2′-F-RNA	300 ng μL^−1^ KOD DSLNK	55 °C, 90 min	7 mM spermidine
2′-NH_2_-RNA and 2′-F-RNA	300 ng μL^−1^ KOD DSLNK	55 °C, 90 min	11 mM cadaverine
2′-NH_2_-RNAand 2′-F-RNA	300 ng μL^−1^ KOD DSLNK	55 °C, 90 min	12 mM putrescine
2′-OMe-RNA	300 ng μL^−1^ KOD DGLNK	72 °C, 10 min	3 mM spermidine
2′-OMe-RNA	300 ng μL^−1^ KOD DGLNK	72 °C, 10 min	5 mM cadaverine
2′-OMe-RNA	300 ng μL^−1^ KOD DGLNK	72 °C, 10 min	8 mM putrescine
DNA (RT from 2′-OMe-RNA)	10 ng μL^−1^ KOD DLK	72 °C, 30 min	7 mM putrescine

**Fig. 2:**
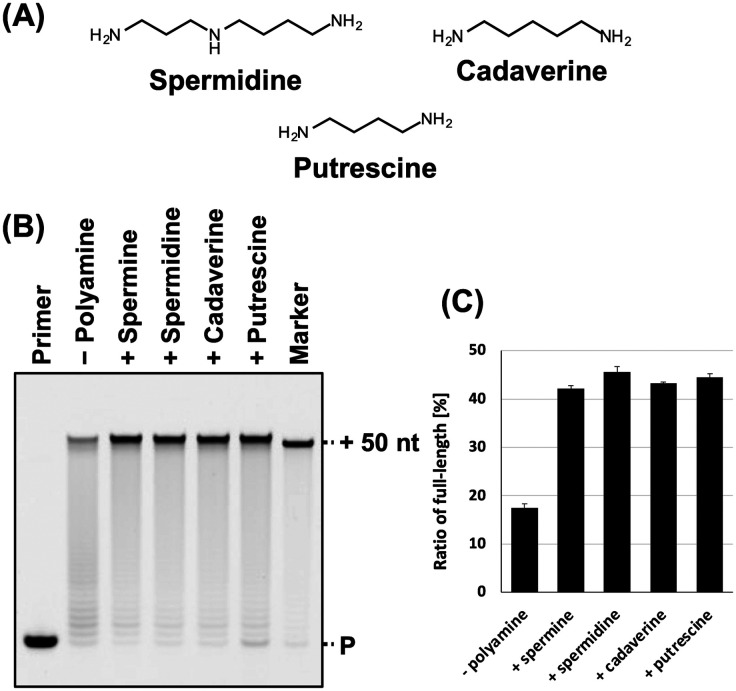
Promotion of ANA synthesis by polyamines. (A) Chemical structure of spermidine, cadaverine, and putrescine. (B) Denaturing PAGE analysis of ANA synthesis by KOD DLK with and without polyamines. The reaction solutions contain optimized concentration of each polyamine (spermine: 5 mM; spermidine: 9 mM; cadaverine: 13 mM; and putrescine: 14 mM). (C) Bar plot of the ratio of full-length product from the reaction shown in Fig. 2B. The ratio of full-length product was calculated as above.

Next, we evaluated the synthesis of ANA using other typical polyamines, including spermidine, cadaverine, and putrescine (Fig. 2A) that promote the DNA synthesis.^31,32^ Each polyamine differs in the number of amino groups and carbon chain length. Spermine, spermidine, cadaverine, and putrescine have four, three, two, and two amino groups, respectively, and their carbon chain length varies as follows: spermine > spermidine > cadaverine > putrescine. The optimal concentration of each polyamine for ANA synthesis using KOD DLK was determined (Fig. S2, ESI†). The optimal concentrations of spermidine, cadaverine, and putrescine were 9, 13, and 14 mM, respectively. The optimal concentration of polyamines for ANA synthesis and were inversely proportional to cation valence of the polyamines. The effects of all the polyamines in ANA synthesis promotion were similar (Fig. 2B and C).

**Fig. 3:**
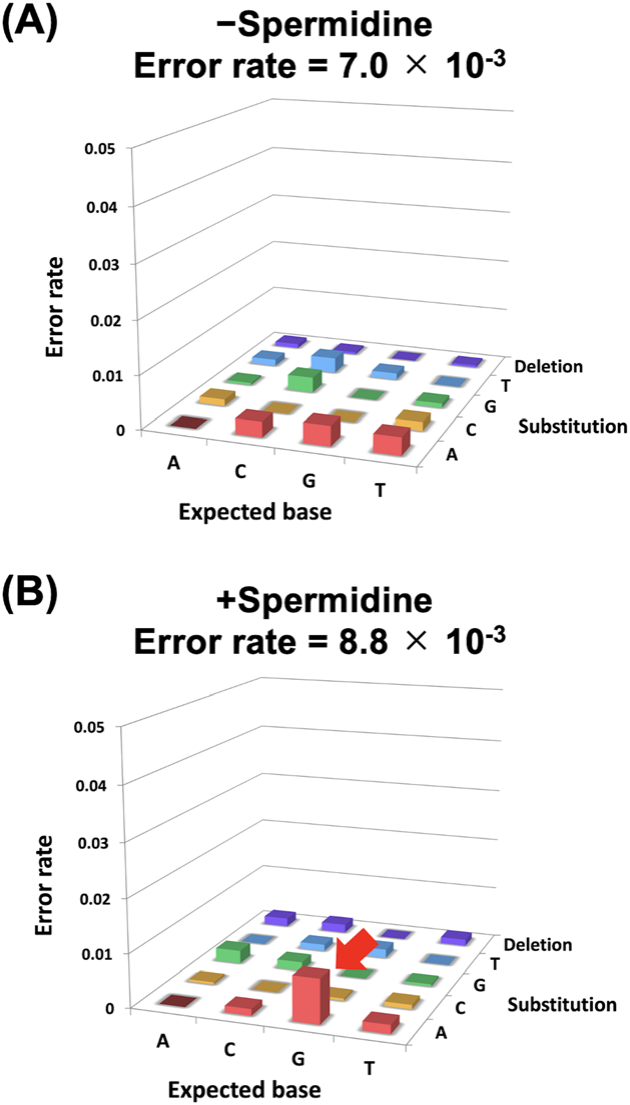
Evaluation of the effect of polyamine on fidelity of ANA synthesis. (A) Error rate profile of both ANA transcription (−spermidine) and reverse transcription (−spermidine) by KOD DL and KOD QDLK, respectively. (B) Error rate profile of both ANA transcription (+spermidine) and reverse transcription (−spermidine) by KOD DL and KOD QDLK, respectively.

We also evaluated the effects of polyamines on the fidelity of ANA synthesis. ANA was synthesised in the presence or absence of spermidine using a single-sequence template DNA. Then, DNA was synthesised in the absence of spermidine using the synthesised ANA strand as the template by KOD QDLK (KOD: V93Q/N210D/A485L/E664K). V93Q is a mutation that inhibits the binding of uracil to the pocket of the N-terminal domain and is effective when using templates containing uracil.^41^ The synthesised DNA that underwent transcription and reverse transcription was analysed using next-generation sequencing, and error rates were calculated. The error rate for each base substitution relative to the expected base is shown in Fig. 3. The addition of spermidine increased G-to-A mutations (Fig. 3B, red arrow). This indicates that instead of Ara-CTP, Ara-UTP was incorporated on the opposite side of the guanine in the template DNA. It is presumed that polyamines stabilised the dG-araU wobble base pair, resulting in the synthesis of a full-length ANA containing the wobble base pair. However, there was only a small difference in fidelity between the reaction conditions with (error rate = 8.8 × 10^−3^) and without spermidine (error rate = 7.0 × 10^−3^). Therefore, effect of polyamines on fidelity did not significantly affect the development of XNA aptamers and XNAzymes.

**Fig. 4:**
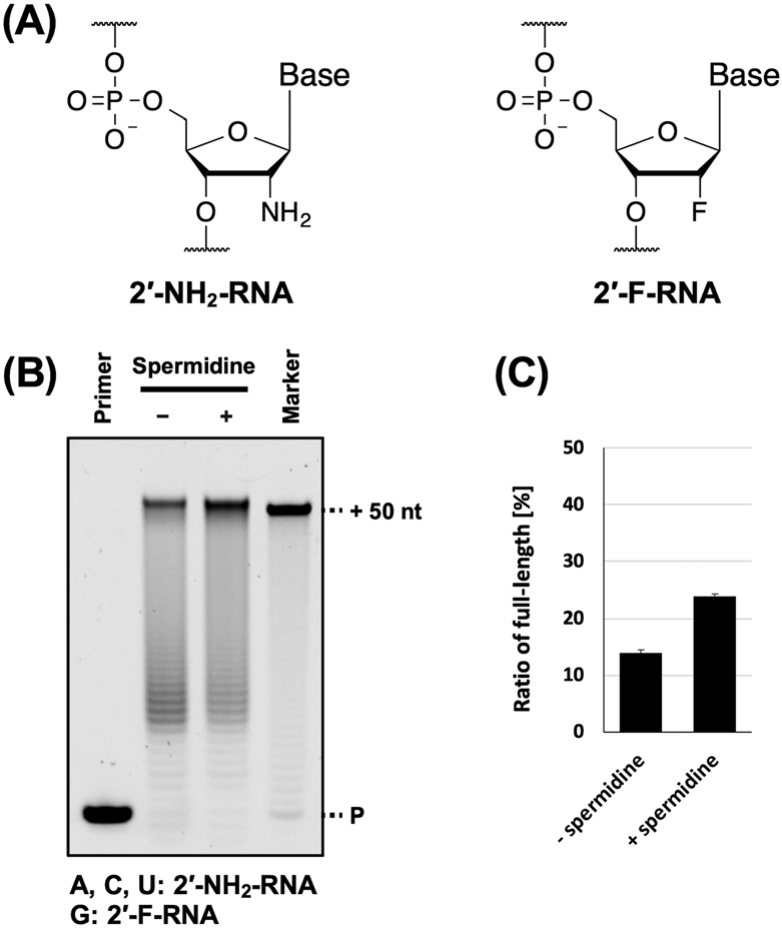
Promotion of 2′-NH_2_-RNA and 2′-F-RNA mixture synthesis by polyamine. (A) Chemical structure of 2′-NH_2_-RNA and 2′-F-RNA. (B) Denaturing PAGE analysis of 2′-NH_2_-RNA and 2′-F-RNA mixture synthesis by KOD DSLNK with and without spermidine. The reaction solutions contain 1 × KOD Dash buffer, 1 mM MnSO_4_, 0.4 μM FAM-labeled DNA primer (25-mer, Primer#1), 0.6 μM DNA template (75-mer, Template#1_N50), 0.1 mM triphosphate mixture (2′-NH_2_-ATP, 2′-NH_2_-CTP, 2′-NH_2_-UTP, and 2′-F-GTP), spermidine (0 or 7 mM), and 300 ng μL^−1^ KOD DSLNK. 2′-NH_2_-RNA and 2′-F-RNA mixture was synthesized at 55 °C for 90 min. (C) Bar plot of the ratio of full-length product from the reaction shown in Fig. 4B. The ratio of full-length product was calculated as above.

Next, we evaluated the utility of polyamines in the synthesis of other XNAs. We used 2′-NH_2_-RNA (Fig. 4A), which has been used in the development of aptamers.^9,10,16^ There is a report on the synthesis of a mixture of 2′-NH_2_-pyrimidine and RNA-purine by T7 RNA polymerase with spermidine.^9^ In addition, 2′-NH_2_-RNA and DNA mixture was synthesised by thermophilic polymerase mutants^37^ without polyamines. In both studies, natural nucleic acids were used for the reactions. Therefore, we aimed to synthesise fully modified nucleic acids using 2′-NH_2_ triphosphates and polyamines. However, the ability of 2′-NH_2_-RNA to form duplexes with DNA is significantly lower.^38^ Therefore, we also used 2′-F-RNA (Fig. 4A), which has been used for aptamer development^11,12,16^ and forms stable duplexes with DNA.^39^ To synthesise the 2′-NH_2_-RNA and 2′-F-RNA mixture, we used a triphosphate mix (2′-NH_2_-ATP, 2′-NH_2_-CTP, 2′-NH_2_-UTP, and 2′-F-GTP) and screened for polymerase mutants. We found that the polymerase mutant KOD DSLNK (KOD: N210D/Y409S/A485L/D614N/E664K) could synthesise the 2′-NH_2_-RNA and 2′-F-RNA mixture. The Y409 residue mutation reduces the steric hindrance of 2′-modified nucleic acid triphosphates,^4,35^ and D614N was designed to improve affinity with the DNA/XNA duplex by anionic residue substitution.^5^ Four polyamines were used for 2′-NH_2_-RNA and 2′-F-RNA mixture synthesis, and all of them promoted 2′-NH_2_-RNA and 2′-F-RNA mixture synthesis (Fig. S3, ESI†). Even when several XNAs were used in combination, the effects of polyamines on XNA synthesis were positive. Moreover, unlike ANA, a preference for polyamines was observed. Among the polyamines, spermidine exhibited a slightly stronger XNA synthesis promoting effect (Fig. 4B and C). In the ANA synthesis, the reaction was believed to almost reach the plateau, and no significant difference was observed.

**Fig. 5:**
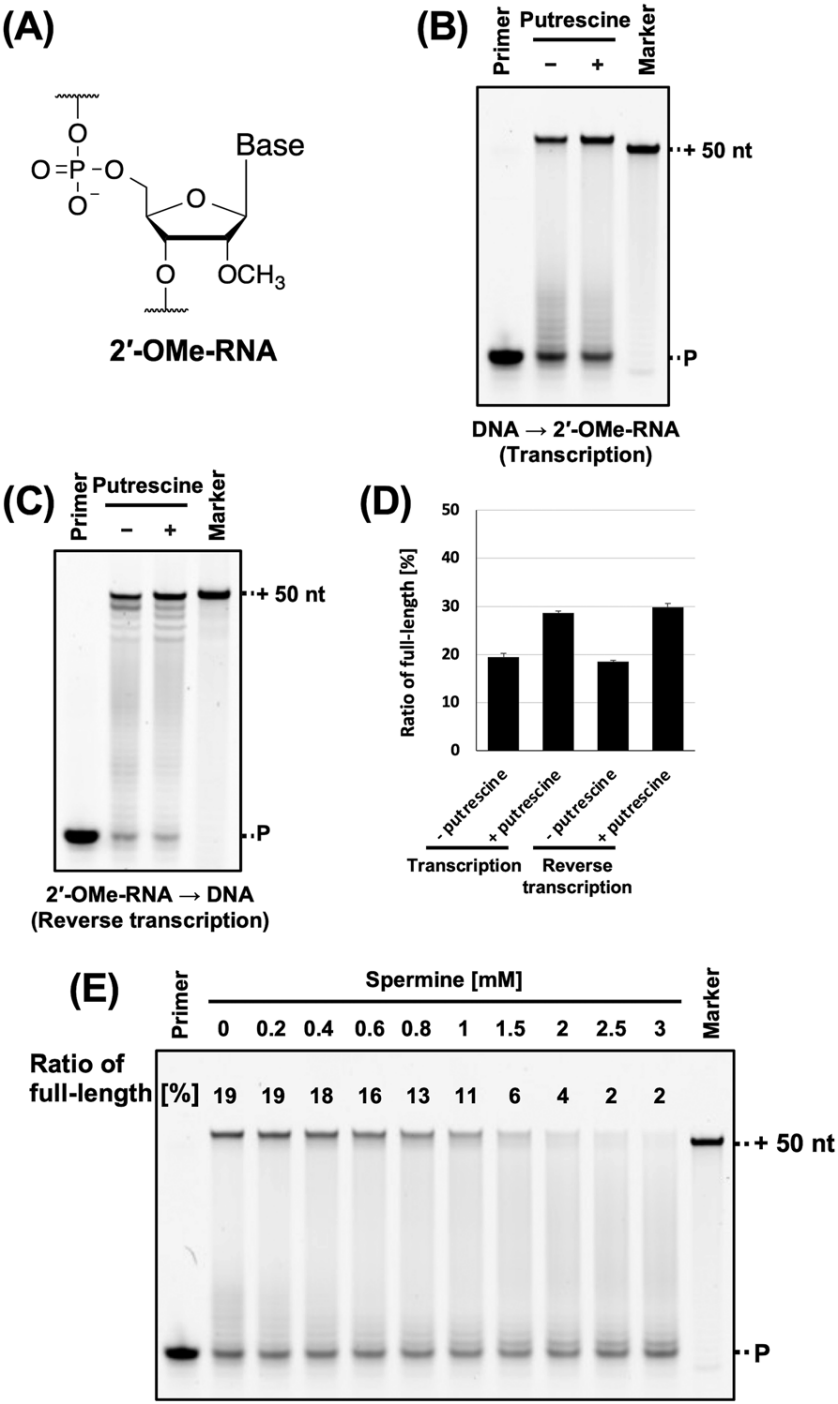
Promotion of 2′-OMe-RNA synthesis by polyamine. (A) Chemical structure of 2′-OMe-RNA. (B) Denaturing PAGE analysis of 2′-OMe-RNA synthesis by KOD DSLNK with and without putrescine. The reaction solutions contain 1 × KOD Dash buffer, 1 mM MnSO_4_, 0.4 μM FAM-labeled 2′-OMe-RNA primer (25-mer, Primer#2_OMe), 0.6 μM DNA template (75-mer, Template#1_N50), 0.1 mM 2′-OMe-NTP, putrescine (0 or 8 mM), and 300 ng μL^−1^ KOD DGLNK. 2′-OMe-RNA was synthesized at 72 °C for 10 min. The ratio of full-length product was calculated as above. (C) Denaturing PAGE analysis of DNA synthesis from 2′-OMe-RNA template by KOD DLK. The reaction solutions contain 1 × KOD Dash buffer, 1 mM MnSO_4_, 0.4 μM HEX-labeled DNA primer (20-mer, Primer#3), 0.6 μM 2′-OMe-RNA template (70-mer, Template#2_OMe) containing 30-mer random sequence, 0.1 mM dNTP, putrescine (0 or 7 mM), and 10 ng μL^−1^ KOD DLK. DNA was synthesized at 72 °C for 30 min. (D) Bar plot of the ratio of full-length product from the reaction shown in Fig. 5B and C. The ratio of full-length product was calculated as above. (E) Optimization test of the concentration of spermine for 2′-OMe-RNA synthesis. Final concentration 0.2–3 mM of spermine was added to the reaction solutions. The ratio of full-length product is above each band.

2′-OMe-RNA (Fig. 5A) is one of the classical modified RNA and owing to its nuclease resistance, has been used in the development of aptamers.^5,9^ However, the stability of 2′-OMe-RNA/DNA duplex is lower than that of 2′-OMe-RNA/RNA duplex.^40^ Therefore, we evaluated the effects of polyamines on 2′-OMe-RNA synthesis. We previously synthesised 2′-OMe-RNA using KOD DGLNK.^5^ We used FAM-labelled 2′-OMe-RNA primer, 2′-OMe triphosphates (2′-OMe-ATP, 2′-OMe-GTP, 2′-OMe-CTP, and 2′-OMe-UTP), KOD DGLNK, and polyamines for synthesising 2′-OMe, and evaluated the effects of polyamines on 2′-OMe-RNA synthesis. We found that spermidine, cadaverine, and putrescine promoted 2′-OMe-RNA synthesis (Fig. S4, ESI†). Among these, putrescine exhibited the strongest effect (Fig. 5B and D). However, spermine did not promote 2′-OMe-RNA synthesis under all conditions examined (Fig. 5E). This is probably due to the competitive inhibition of KOD DGLNK by spermine. Since polyamines and polymerases both bind to nucleic acid duplexes, they may compete for binding to nucleic acid duplexes (Fig. S7, ESI†). The optimal concentration of polyamines for XNA synthesis may be determined by the balance between the benefit of stabilising the DNA/XNA duplex and the disadvantage of competitive inhibition of polymerases. Spermine has a longer carbon chain and higher cation valence than the other polyamines and binds strongly to duplex; therefore, it exerts a stronger competitive inhibitory effect on polymerase. On the other hand, due to steric hindrance, nucleic acid modification weakens the interaction between the polymerase and the duplex. Thus, with the use of spermine, the benefits of stabilising the 2′-OMe-RNA/DNA duplex may not outweigh the disadvantages of the competitive inhibition of KOD DGLNK by spermine. If spermine could not promote XNA synthesis, using another polyamine would be an effective solution.

*In vitro* selection using XNA libraries requires DNA synthesis from the XNA templates (reverse transcription). The synthesised cDNA is amplified by polymerase chain reaction (PCR) and used as a template for the next round of XNA synthesis. Promoting DNA synthesis from XNA templates will prevent missing active sequences during *in vitro* selection and enable better selection of XNA aptamers and XNAzymes. Therefore, we evaluated the effect of polyamine on DNA synthesis from the 2′-OMe-RNA template containing random sequences. Previously, we found that KOD DLK effectively synthesises DNA from 2′-OMe-RNA.^5^ In this study, DNA synthesis from the 2′-OMe-RNA template was promoted by polyamine (Fig. 5C, D and Fig. S5, ESI†).

Polyamines promoted the XNAs synthesis and reverse transcription by increasing the stability of the DNA/XNA duplex. In addition, the polyamines had negligible effect on the fidelity of XNA synthesis. The optimal polyamine concentration depended on XNA and polymerase mutants. We believe that the optimal polyamine concentration for XNA synthesis also depend on several other factors. For example, because polyamines and polymerase mutants compete for nucleic acids, the concentration of polymerase mutants may affect the concentration of polyamines required for optimal XNA synthesis. In addition, the reaction temperature in XNA synthesis may affect the optimal concentration of polyamines because the affinities between polyamines and nucleic acids as well as between polymerases and nucleic acids change with a change in temperature. Therefore, it is crucial to select a polyamine whose concentration can be optimised in each case.

The advantage of polyamines is their duplex stabilisation. Therefore, when using XNA that forms stable duplexes with DNA at high temperatures, polyamines either do not or only marginally promote XNA synthesis. Therefore, the need for polyamines is dependent on the properties of XNA used. Another advantage of polyamines, we believe, is the ability to regulate the strength of the interaction between the polymerase mutant and the DNA/XNA duplex. Too strong interaction between the polymerase and the nucleic acid duplex caused by the mutation slows down the movement of the polymerase on the nucleic acid during the extension reaction. In fact, DNA synthesis from DNA templates by KOD DLK, containing the E664K mutation, which enhances the interaction between the polymerase and nucleic acids, was less efficient than that by KOD DL (data not shown). Presumably, during KOD DLK-driven ANA synthesis, polyamines reduced the excessive interaction between KOD DLK and the DNA/ANA duplex, thereby promoting ANA synthesis. However, if the interaction between polymerase mutants and DNA/XNA is too weak, the benefits offered by polyamines may be reduced. Therefore, whether the regulation by reducing interaction between polymerase mutants and DNA/XNA has a positive effect depends on XNA and polymerase mutants used.

## Conclusions

Promoting XNAs synthesis by polyamines is a versatile method. Optimal polyamine selection and optimization of polyamine concentration enable efficient XNA synthesis. Promoting the synthesis efficiency of various XNAs will improve the diversity of nucleic acid libraries and aid in the development of XNA aptamers and XNAzymes.

## Author contributions

Conceptualization, H. H.; investigation, H. H.; data curation, H. H.; writing—original draft preparation, H. H.; writing—review and editing, Y. K., and S. O.; visualization, H. H.; supervision, Y. K. and S. O.; project administration, Y. K. and S. O.; funding acquisition, H. H., and Y. K. All authors have read and agreed to the published version of the manuscript.

## Conflicts of interest

There are no conflicts to declare.

## Supplementary Material

CB-005-D4CB00017J-s001
