# Supplementary material for: Polyamines promote xenobiotic nucleic acid synthesis by modified thermophilic polymerase mutants
Source: RSC Chem Biol. 2024 Apr 4;5(5):467–72. doi: 10.1039/d4cb00017j (PMC11078213; doi:10.1039/d4cb00017j)
Supplement: CB-005-D4CB00017J-s001 [file CB-005-D4CB00017J-s001.pdf]

Electronic Supplementary Material (ESI) for RSC Chemical Biology.
This journal is © The Royal Society of Chemistry 2024

Supplementary Material for:

Polyamines promotes xenobiotic nucleic acid synthesis by modified thermophilic polymerase variants

Hidekazu Hoshino,*[a] Yuuya Kasahara,[a,b] and Satoshi Obika*[a,b]

[a] National Institutes of Biomedical Innovation, Health and Nutrition (NIBIOHN), 7-6-8 Saito-Asagi, Ibaraki 567-0085, Osaka, Japan

[b] Graduate School of Pharmaceutical Sciences, Osaka University, 1-6 Yamadaoka, Suita 565-0871, Osaka, Japan

*Corresponding Author: h-hoshino@nibiohn.go.jp (H. Hoshino); obika@phs.osaka-u.ac.jp (S. Obika)

Outline:

1. Supplementary figures

Figure S1. Optimization of spermine concentration for ANA synthesis.

Figure S2. Optimization of spermidine, cadaverine, and putrescine concentrations for ANA synthesis.

Figure S3. Optimization of spermine, spermidine, cadaverine, and putrescine concentrations for 2′-NH_2-RNA and 2′-F-RNA mixture synthesis.

Figure S4. Optimization of spermidine, cadaverine, and putrescine concentrations for 2′-OMe-RNA synthesis.

Figure S5. Optimization of putrescine concentration for DNA synthesis from 2′-OMe-RNA template.

Figure S6. Promotion of ANA synthesis by spermidine in the absence of Mn^{2+}.

Figure S7. Evaluation of inhibitory effect of polyamines for XNA synthesis.

2. Supplementary table

Table S1. Oligonucleotide sequences

3. Materials and Methods

4. References

Contents:

1. Supplementary figures

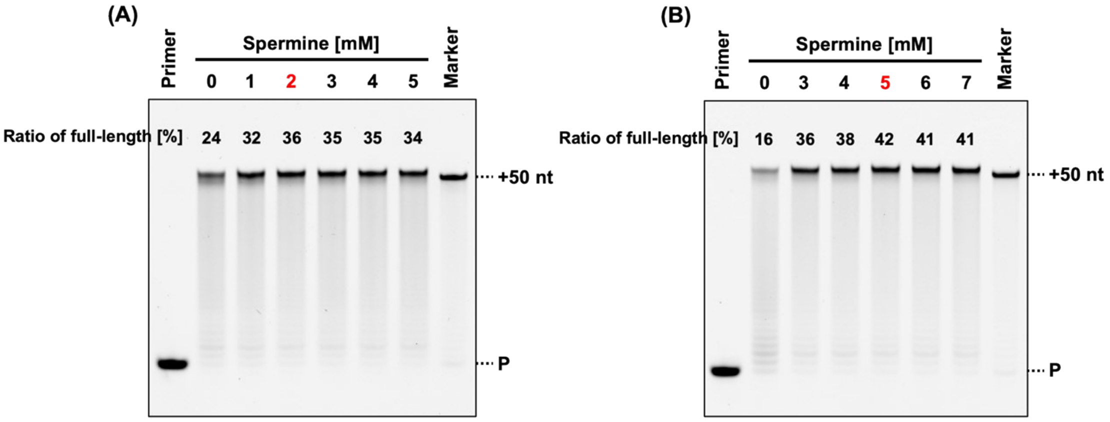

Figure S1. Optimization of spermine concentration for ANA synthesis. (A), (B) Denaturing PAGE analysis of ANA synthesis by KOD DL (A) or KOD DLK (B) with spermine. Reaction solution contains 1×KOD Dash buffer, 1 mM $MnSO_4$, 0.4 µM FAM-labeled DNA primer (25-mer, Primer#1), 0.6 µM DNA template (75-mer, Template#1_N50), 0.1 mM Ara-NTP, spermine (1–5 mM for KOD DL; 3–7 mM for KOD DLK), and 20 ng/µL KOD DL or KOD DLK. ANA was synthesized at 60 °C for 5 min. The ratio of full-length product is above each band. The optimal concentration (red text) was determined based on the ratio of full-length product. Marker was synthesized by primer extension using dNTP.

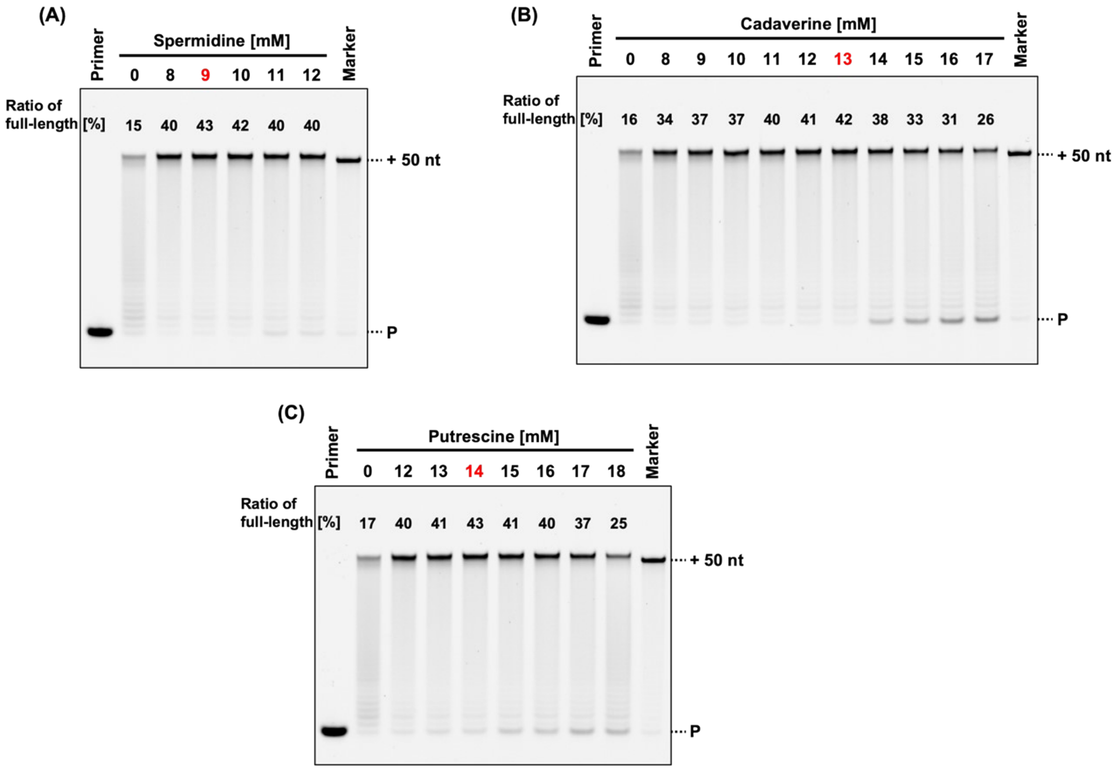

Figure S2. Optimization of spermidine, cadaverine, and putrescine concentrations for ANA synthesis. (A)–(C) Denaturing PAGE analysis of ANA synthesis by KOD DLK with spermidine (A), cadaverine (B), and putrescine (C). Reaction solutions contain polyamine ((A): 8–12 mM spermidine; (B): 8–17 mM cadaverine; and (C): 12–18 mM putrescine) and the same components as Figure S1(B) except for spermine. ANA was synthesized at 60 °C for 5 min. The optimal concentration (red text) was determined based on the ratio of full-length product.

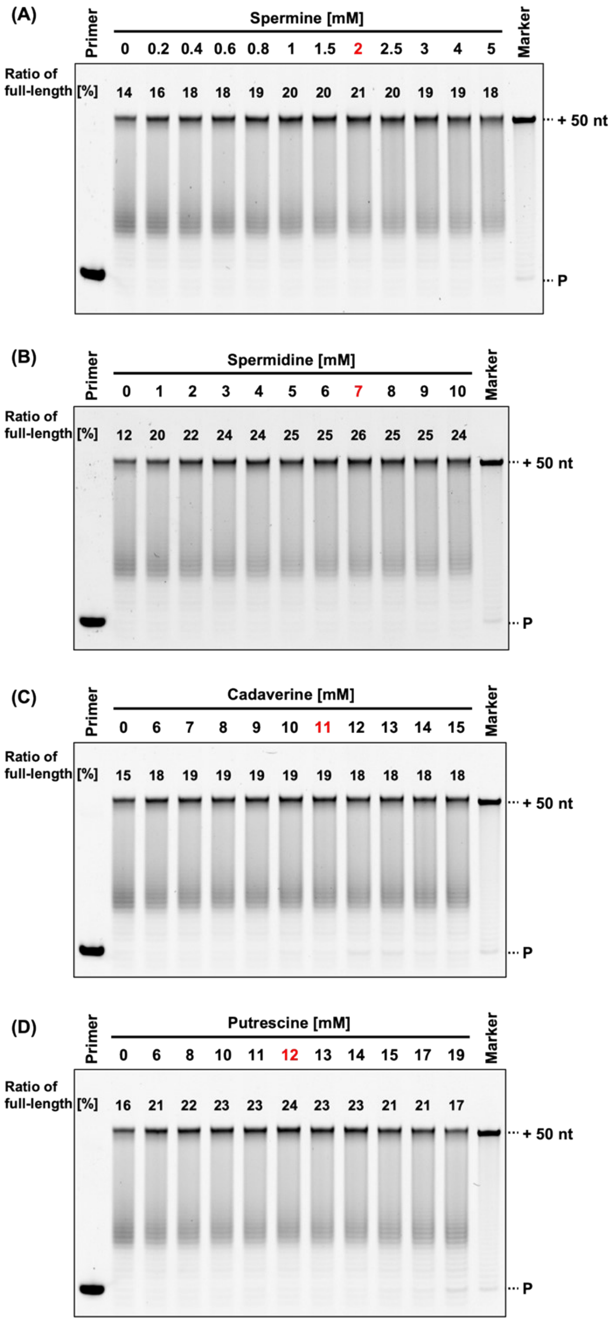

Figure S3. Optimization of spermine, spermidine, cadaverine, and putrescine concentrations for 2′-NH_2-RNA and 2′-F-RNA mixture synthesis. (A)–(D) Denaturing PAGE analysis of 2′-NH_2-RNA and 2′-F-RNA mixture synthesis by KOD DSLNK with spermine (A), spermidine (B), cadaverine (C), and putrescine (D). Reaction solution contains 1×KOD Dash buffer, 1 mM $MnSO_4$, 0.4 μM FAM-labeled DNA primer (25-mer, Primer#1), 0.6 μM DNA template (75-mer, Template#1_N50), 0.1 mM triphosphate mixture (2′-NH_2-ATP, 2′-NH_2-CTP, 2′-NH_2-UTP, and 2′-F-GTP), polyamine ((A): 0.2–5 mM spermine; (B): 1–10 mM spermidine; (C): 6–15 mM cadaverine; and (D): 6–19 mM putrescine), and 300 ng/μL KOD DSLNK. 2′-NH_2-RNA and 2′-F-RNA mixture was synthesized at 55 °C for 90 min. The optimal concentration (red text) was determined based on the ratio of full-length product.

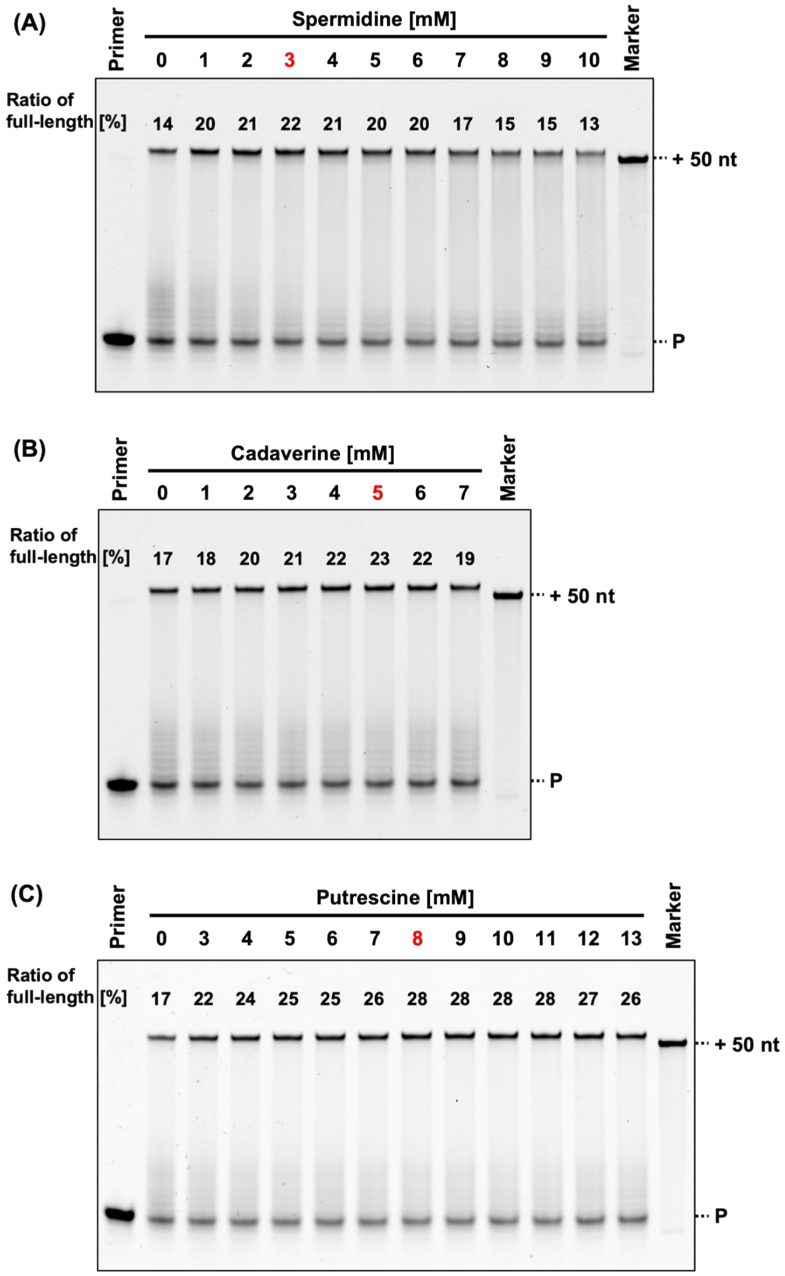

Figure S4. Optimization of spermidine, cadaverine, and putrescine concentrations for 2′-OMe-RNA synthesis. (A)–(C) Denaturing PAGE analysis of 2′-OMe-RNA synthesis by KOD DGLNK with (A) spermidine, (B) cadaverine, and (C) putrescine. Reaction solution contains 1×KOD Dash buffer, 1 mM $MnSO_4$, 0.4 µM FAM-labeled 2′-OMe-RNA primer (25-mer, Primer#2_OMe), 0.6 µM DNA template (75-mer, Template#1_N50), 0.1 mM 2′-OMe-NTP, polyamine ((A): 0.2–3 mM spermine; (B): 1–10 mM spermidine; (C): 1–7 mM cadaverine; and (D): 3–13 mM putrescine), and 300 ng/µL KOD DGLNK. 2′-OMe-RNA was synthesized at 72 °C for 10 min. The optimal concentration (red text) was determined based on the ratio of full-length product.

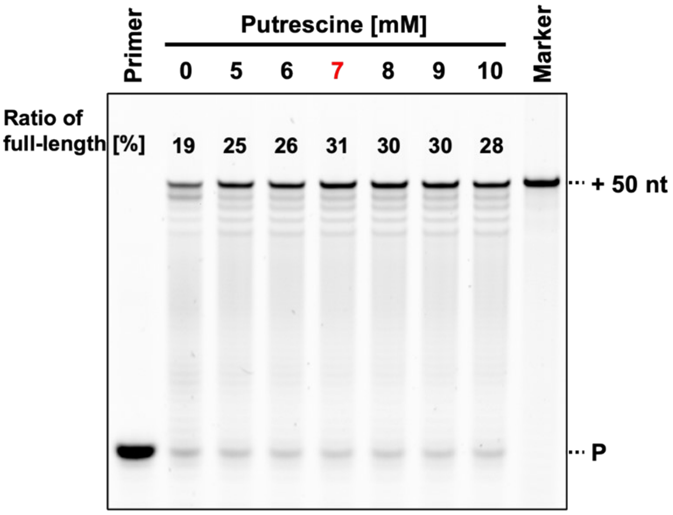

Figure S5. Optimization of putrescine concentration for DNA synthesis from 2′-OMe-RNA template. Denaturing PAGE analysis of DNA synthesis by KOD DLK with putrescine. Reaction solution contains 1×KOD Dash buffer, 1 mM $MnSO_4$, 0.4 μM HEX-labeled DNA primer (20-mer, Primer#3), 0.6 μM 2′-OMe-RNA template (70-mer, Template#2_OMe), 0.1 mM dNTP, 5–10 mM putrescine, and 10 ng/μL KOD DLK. DNA was synthesized at 72 °C for 30 min. The optimal concentration (red text) was determined based on the ratio of full-length product.

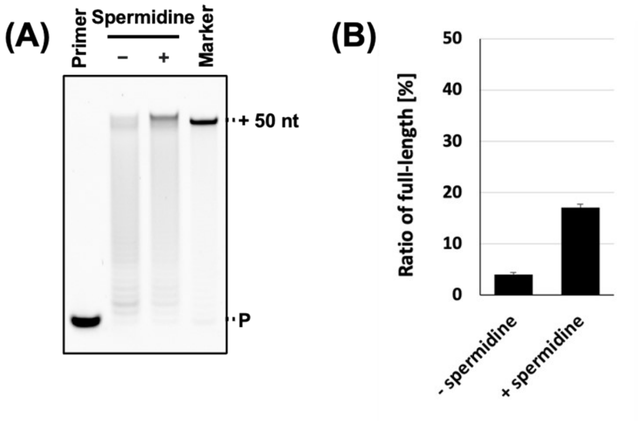

Figure S6. Promotion of ANA synthesis by spermidine in the absence of Mn^{2+}. (A) Denaturing PAGE analysis of ANA synthesis by KOD DLK with spermidine. Reaction solution contains 1×KOD Dash buffer containing 1.2 mM Mg^{2+}, 0.4 μM FAM-labeled DNA primer (25-mer, Primer#1), 0.6 μM DNA template (75-mer, Template#1_N50), 0.1 mM Ara-NTP, 0 or 9 mM spermidine, and 20 ng/μL KOD DLK. ANA was synthesized at 60 °C for 30 min. (B) Bar plot of the ratio of full-length product from the reaction shown in Figure S6A. The ratio of full-length product was calculated from the fluorescence intensity. Data represent the mean and standard error of three independent experiments.

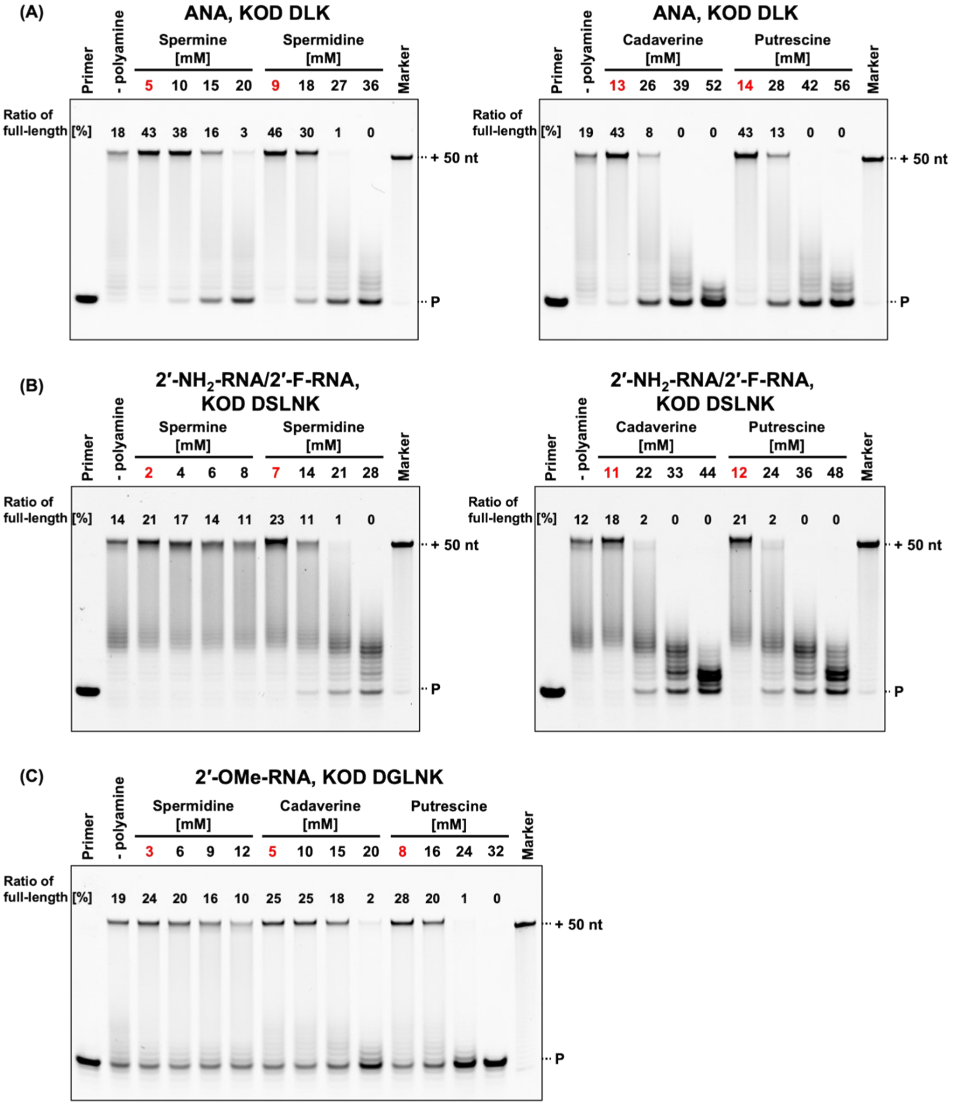

Figure S7. Evaluation of inhibitory effect of polyamines for XNA synthesis. (A) Denaturing PAGE analysis of ANA synthesis by KOD DLK with 5–20 mM spermine, 9–36 mM spermidine, 13–52 mM cadaverine, or 14–56 mM putrescine. (B) Denaturing PAGE analysis of 2′-NH_2-RNA and 2′-F-RNA mixture synthesis by KOD DSLNK with 2–8 mM spermine, 7–28 mM spermidine, 11–44 mM cadaverine, or 12–48 mM putrescine. (C) Denaturing PAGE analysis of 2′-OMe-RNA synthesis by KOD DGLNK with 3–12 mM spermidine, 5–20 mM cadaverine, or 8–32 mM putrescine. The reaction conditions for each were the same as above except for the concentration of polyamines. The optimal concentration is indicated in red text.

2. Supplementary table

Table S1. Oligonucleotide sequences

2′-OMe-RNA is shown in blue. Mutation site is shown in red. Sequence for fidelity analysis is shown in green.

Name	Sequence	Supplier
Primer#1	5′-FAM-GGATTAGCGAACAGGCCATACCTTT-3′	Thermo Fisher Scientific
Template#1_N50	5′-<br>NNNNNNNNNNNNNNNNNNNNNNNNNNNNNNNNN<br>NNNNNNNNNNNNNNNNNAAAGGTATGGCCTGTTCGCT<br>AATCC-3′ (N = A, G, C, and T mix)	Ajinomoto Bio-Pharma
Primer#2_OMe	5′-FAM-GGAUUAGCGAACAGGCCAUACCUUU-3′	Japan Bio Services
Primer#3	5′-ATGGCAGCCACAGCGATTTC-3′	Thermo Fisher Scientific
Template#2_OMe	5′-<br>UCGCCUUGCCGGAUCGCAGANNNNNNNNNNNNNN<br>NNNNNNNNNNNNNNNGAAAUCGCUGUGGCUGCCAU<br>-3′ (N = A, G, C, and U mix)	Japan Bio Services
Template#3	5′-<br>TAATCTCACTACGACTCGACACAACTGGATAGTTCA<br>AGCTTGCCCGATACGGTCCCTATGCGTTGTTATCTGA<br>AAGAAAAAAAA-Biotin-3′	Japan Bio Services
Primer#4	5′-<br>CAATAGCGAGCAGAGCATACTTTCAGATAGCAACGC<br>ATAGG-3′	Ajinomoto Bio-Pharma
Primer#5	5′-Biotin-TAATCTCACTACGACTCGACA-3′	Ajinomoto Bio-Pharma
Primer#6	5′-TAATCTCACTACGACTCGACA-3′	Thermo Fisher Scientific
Primer#7	5′-CAATAGCGAGCAGAGCATAC-3′	Ajinomoto Bio-Pharma

3. Materials and Methods.

Materials. All reagents were purchased from commercial suppliers. ANA triphosphates (Ara-ATP, Ara-GTP, Ara-CTP, and Ara-UTP), 2′-NH_2 triphosphates (2′-NH_2-ATP, 2′-NH_2-CTP, 2′-NH_2-UTP), 2′-F-GTP, and 2′-OMe triphosphates (2′-OMe-ATP, 2′-OMe-GTP, 2′-OMe-CTP, and 2′-OMe-UTP) were obtained from TriLink Bio Technologies (San Diego, CA, USA). Oligonucleotides were synthesized by Thermo Fisher Scientific Japan (Tokyo, Japan), Ajinomoto Bio-Pharma (Osaka, Japan), or Japan Bio Services (Saitama, Japan). Thermal cycler WK-0518 (Wako, Osaka, Japan) was used for reaction. The gel electrophoresis system Mini-PROTEAN Tetra Cell (Bio-Rad, Hercules, CA, USA) was used for denaturing PAGE. The MiSeq system and MiSeq Reagent Kit Nano v2 (300 Cycle) (Illumina, San Diego, CA, USA) were used for next-generation sequencing (NGS).

Production and purification of polymerase mutants. Polymerase mutants were produced and purified in the same manner as previously reported[1]. The following is a brief description. The KOD polymerase gene was inserted into NdeI and SalI site of pET-49b (+) (Merck Millipore, Billerica, MA, USA). The mutations were introduced by PrimeSTAR Mutagenesis Basal Kit (TAKARA Bio, Shiga, Japan). The KOD polymerase mutants were produced in *E. coli* BL21(DE3) by induction of gene expression with IPTG at 30 °C for 6 h. After sonication, the KOD polymerase mutants were prepared by removing insolubilized proteins by heating (80 °C, 15 min) and purification through HiTrap Heparin HP column (GE Healthcare, Chicago, IL, USA). Finally, the buffer was replaced by stock solution [50 mM Tris-HCl (pH 8.0), 50 mM KCl, 1 mM DTT, 0.1% Tween 20, 0.1% Nonidet-P40 and 50% (v/v) glycerol], and polymerase mutants were stored at -20 °C.

Primer extension. All samples were sequentially added polyamine and polymerase mutants after primer annealing. The 10 μL of reaction solutions were incubated for nucleic acid synthesis, and 2 μL of stop solution (40 mM EDTA and 0.1% Bromophenol blue) and 12 μL of 10 M urea were immediately added to the reaction solutions. The sample solutions were denatured by boiling at 95 °C for 5 min and used for denaturing PAGE at 55 °C. The gel images were acquired by Chemi-Doc (Bio-Rad) and analyzed by Image Lab software (Bio-Rad). The reaction conditions for each nucleic acid are described below. **ANA synthesis**: The reaction mixture [1×KOD Dash buffer (TOYOBO, Osaka, Japan), 1 mM $MnSO_4$, 0.4 μM FAM-labeled DNA primer (Primer#1), 0.6 μM DNA template (Template#1_N50), 0.1 mM Ara-NTP (Ara-ATP, Ara-GTP, Ara-CTP, and Ara UTP), polyamine (1–7 mM spermine, 8–12 mM spermidine, 8–17 mM cadaverine, or 12–18 mM putrescine), and 20 ng/μL polymerase mutant (KOD DL or KOD DLK)] was incubated at 60 °C for 5 min. **2′-NH_2-RNA and 2′-F-RNA mixture synthesis**: The reaction

mixture [1×KOD Dash buffer, 1 mM $MnSO_4$, 0.4 μM DNA primer (Primer#1), 0.6 μM DNA template (Template#1_N50), 0.1 mM Ara-NTP (Ara-ATP, Ara-GTP, Ara-CTP, and Ara UTP), polyamine (0.2–5 mM spermine, 1–10 mM spermidine, 6–15 mM cadaverine, or 6–19 mM putrescine), and 300 ng/μL KOD DSLNK] was incubated at 55 °C for 90 min. **2′-OMe-RNA synthesis**: The reaction mixture [1×KOD Dash buffer, 1 mM $MnSO_4$, 0.4 μM FAM-labeled 2′-OMe-RNA primer (Primer#2_OMe) and 0.6 μM DNA template (Template#1_N50), 0.1 mM 2′-OMe-NTP, polyamine (0.2–3 mM spermine, 1–10 mM spermidine, 1–7 mM cadaverine, and 3–13 mM putrescine), and 300 ng/μL KOD DGLNK] was incubated at 72 °C for 10 min. **DNA synthesis from 2′-OMe-RNA (reverse transcription)**: The reaction mixture [1×KOD Dash buffer, 1 mM $MnSO_4$, 0.4 μM HEX-labeled DNA primer (Primer#3), 0.6 μM 2′-OMe-RNA template (Template#2_OMe), 0.1 mM dNTP, 5–10 mM putrescine, and 10 ng/μL KOD DLK] was incubated at 72 °C for 30 min.

Fidelity assay. The fidelity evaluation procedure is the same as previously reported[1] except for the ANA transcription and reverse transcription conditions. The conditions for ANA transcription and reverse transcription are described below. **ANA transcription**: The reaction mixture [1×KOD buffer#2 (TOYOBO), 1 mM $MnSO_4$, 0.1 μM DNA primer (Primer#4), 0.5 μM biotinylated DNA template (Template#3), 0.2 mM Ara-NTP (Ara-ATP, Ara-GTP, Ara-CTP, and Ara UTP), 5 mM spermidine, and 20 ng/μL KOD DL] (20 μL) was incubated at 60 °C for 10 min. **ANA reverse transcription**: The reaction mixture [1×KOD buffer#2, 1 mM $MnSO_4$, 0.5 μM biotinylated DNA primer (Primer#5), synthesized ANA, 0.2 mM dNTP, and 20 ng/μL KOD QDLK] (10 μL) was incubated at 45 °C for 30 min. The cDNA products were amplified by PCR using primers Primer#6 and Primer#7 and analyzed by NGS. The error rate was determined by counting the number of substitutions, insertions, and deletions in 9,600 bases.

4. References

1. H. Hoshino, Y. Kasahara, M. Kuwahara and S. Obika, *J. Am. Chem. Soc.*, 2020, **142**, 21530–21537.
